# Long-Term Control of Breast Cancer Brain Metastases Using Abemaciclib and Letrozole Combination Therapy: A Case Report

**DOI:** 10.7759/cureus.79805

**Published:** 2025-02-27

**Authors:** Yumi Nozaki, Minori Yamamuro, Noriyoshi Tanaka, Nobuyuki Kamo, Juichiro Konishi

**Affiliations:** 1 Department of Medical Oncology, National Hospital Organization Saitama Hospital, Saitama, JPN; 2 Department of Breast Center, National Hospital Organization Saitama Hospital, Saitama, JPN

**Keywords:** abemaciclib, brain metastases, breast cancer, case report, hormone receptor

## Abstract

Breast cancer brain metastases are among the most common distant metastases and have a poor prognosis. However, the effects of subtype-specific systemic therapy on breast cancer brain metastases are unknown. This report highlights the long-term control of breast cancer brain metastases using abemaciclib and letrozole combination therapy without severe side effects. We report a case of a middle-aged premenopausal female patient who experienced convulsions and was diagnosed with hormone receptor-positive breast cancer brain metastases. After standard-of-care whole-brain radiation therapy, systemic chemotherapy was administered, and the primary tumor and multiple brain metastases were sufficiently reduced. However, treatment was discontinued because peripheral neuropathy worsened. Abemaciclib, letrozole, and luteinizing hormone-releasing hormone agonists were administered as maintenance therapy. The treatment maintained a stable disease and no new lesions were observed. Cyclin-dependent kinase 4/6 inhibitors and endocrine maintenance therapy are effective treatments for breast cancer brain metastases.

## Introduction

Breast cancer is the most common malignancy among women worldwide. The incidence of symptomatic breast cancer brain metastases, which have a poor prognosis, is 5% to 15% during the clinical course [1,2]. One study found that 0.41% of patients had brain metastases at the time of the breast cancer diagnosis [3]. The current standard local therapies for brain metastases include surgical resection, stereotactic radiosurgery, and whole-brain radiotherapy [4]. The effects of systemic therapy vary depending on the permeability of the blood-brain barrier (BBB) on the therapeutic agent, and their entry is predicated on the disruption of the BBB [5]. Subtype-specific systemic therapies have been developed for metastatic breast cancer. The addition of cyclin-dependent kinase (CDK) 4/6 inhibitors to endocrine therapy (ET) has improved the outcomes of patients with hormone receptor-positive (HR+) and human epidermal growth factor receptor 2-negative (HER2−) advanced breast cancer [6,7]. We report a patient with advanced breast cancer and brain metastases who experienced long-term disease maintenance with treatment comprising CDK4/6 inhibitors and ET.

## Case presentation

**Figure 1:**
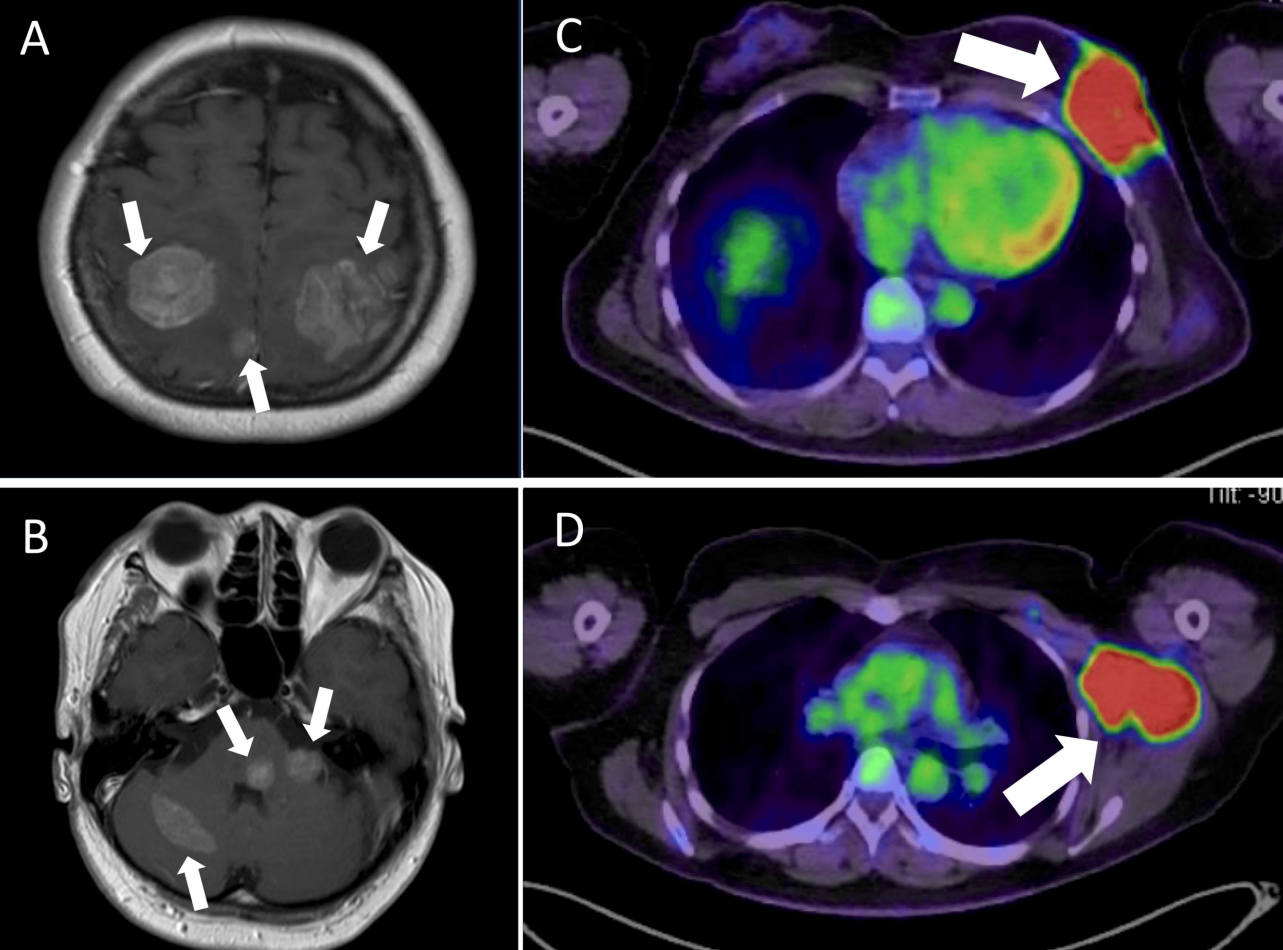
MRI and PET/CT imaging findings. Magnetic resonance imaging (MRI) and positron emission tomography (PET)/computed tomography (CT) findings before treatment with whole-brain radiotherapy. (A) Brain metastases in the cerebrum. (B) Brain metastases in the cerebellum. (C) A 50-mm mass in the left breast. (D) Left axillary lymph nodes.

A middle-aged premenopausal female patient experienced convulsions and was referred to our neurosurgery department. MRI revealed multiple brain metastases in the parietal lobe of the cerebrum and the anterior and posterior lobe of the cerebellum (Figures 1A-1B). Subsequently, 18F-fluorodeoxyglucose positron emission tomography/computed tomography (PET/CT) revealed a 10-cm mass in the left breast and a 6-cm mass in the left axilla lymph node with a high concentration of standardized uptake value (max) 1 hour of 20.5 and 15.8, respectively (Figures 1C-1D); the findings were suggestive of primary breast cancer with nodal involvement. Laboratory data showed an elevated cancer antigen (CA) 15-3 level (61.7 U/mL; reference range ≤ 31.7) and a carcinoembryonic antigen level within normal limits (4.7 U/mL; reference range ≤ 5.0). The patient underwent a needle biopsy of the breast mass, which indicated the diagnosis of invasive ductal carcinoma nuclear grade 1. Results of immunohistochemistry revealed positive estrogen receptor and progesterone receptor and HER2 1+ score (HER2-negative) results; additionally, the Ki-67 index indicated that 50% of the cells were positive (a luminal B-like subtype that was HER2-negative). Therefore, cT4cN2aM1 stage Ⅳ disease was diagnosed. BRCA 1/2 germline gene mutation PCR-based testing yielded negative results.

**Figure 2:**
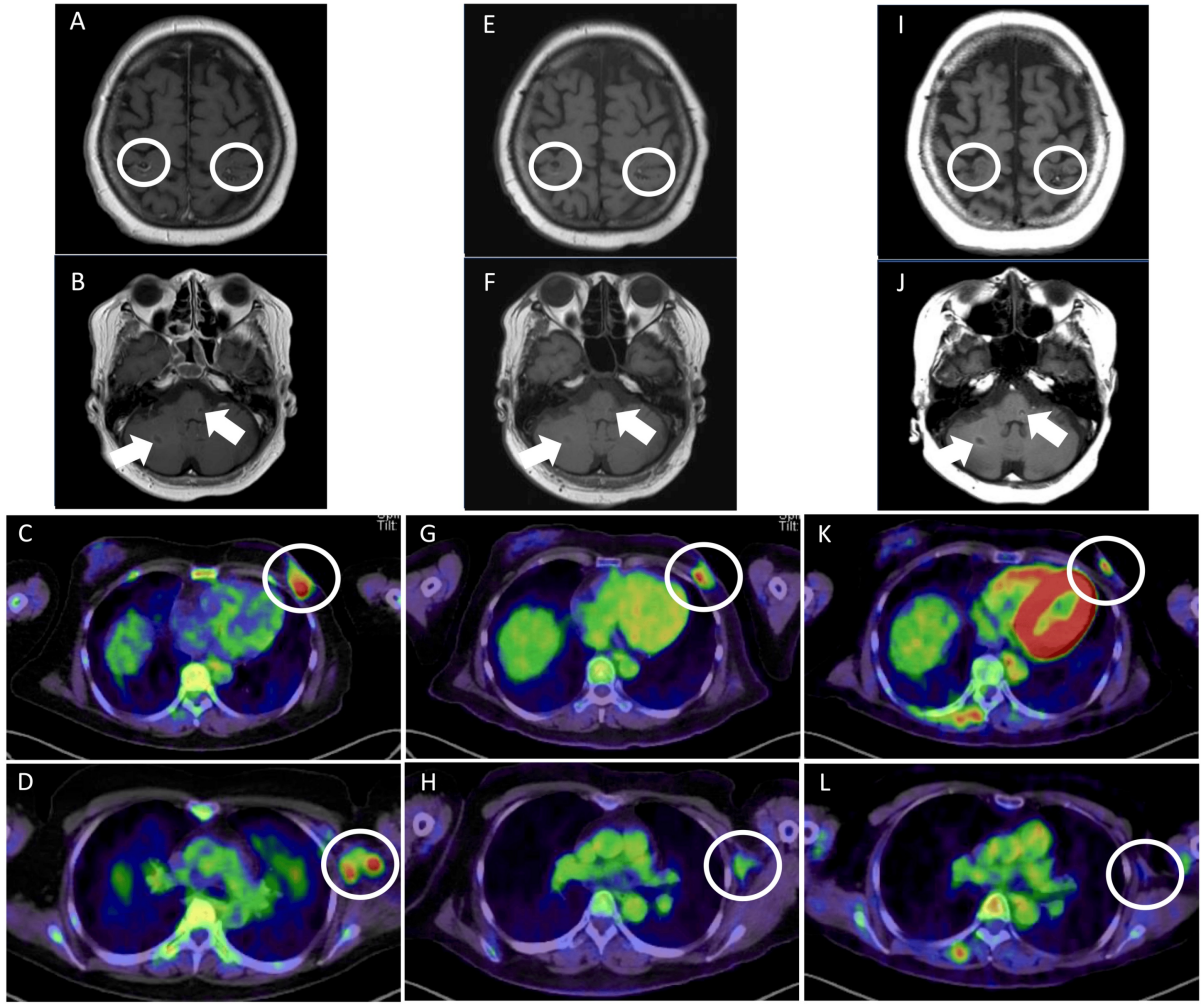
MRI and PET/CT imaging findings Magnetic resonance imaging (MRI) and positron emission tomography (PET)/computed tomography (CT) findings after treatment. (A-D) After treatment with whole-brain radiotherapy (WBRT) and three cycles of epirubicin plus cyclophosphamide (EC), the tumor shrank and disappeared. (E-H) After treatment with bevacizumab and paclitaxel, the therapeutic effect was maintained. (I-L) During treatment with abemaciclib, letrozole, and luteinizing hormone-releasing hormone (LH-RH) agonist, the tumor in the left breast and left axillary lymph nodes continued to reduce, and brain metastases in the cerebrum and cerebellum multiple continued to remain stable. No new lesions were observed.

The patient was classified as recursive partitioning analysis (RPA) class II (for uncontrolled primary tumor) and underwent whole-brain irradiation (30 Gy/10 Fr) for multiple brain metastases. After three cycles of epirubicin plus cyclophosphamide (90 mg/m2 and 600 mg/m2, respectively, every 3 weeks) therapy, MRI showed significant reduction and disappearance of multiple brain metastases, and PET/CT revealed reduction of the primary tumor and that of the left axillary lymph nodes. This response was considered a partial response (Figures 2A-2D). Blood tests revealed that the CA 15-3 level was within normal limits (30.9 U/mL; reference range ≤ 31.7). However, because PET/CT revealed a diffuse distribution of ground-grass opacity in both lungs and drug-induced lung injury was suspected, the treatment was changed.

Her next treatment was changed to bevacizumab (10 mg/kg every 2 weeks) and paclitaxel (days 1, 8, and 15 of a 4-week cycle). Because grade 3 neutropenia and grade 1 peripheral neuropathy occurred, the dose of paclitaxel was reduced to two cycles, and the paclitaxel schedule was changed to biweekly. After five cycles of the treatment, the therapeutic effect was maintained and the disease was considered stable (Figure 2E-2H); however, because grade 2 peripheral neuropathy occurred, treatment was discontinued in favor of abemaciclib (150 mg twice daily), letrozole (2.5 mg daily), and luteinizing hormone-releasing hormone agonist, as a third-line treatment. During the first cycle of treatment, grade 1 diarrhea developed; however, it improved with medication. Because grade 2 neutropenia and grade 1 anemia occurred, the abemaciclib dose was reduced to 50 mg (twice daily) within a 2-month period. More than 3 years have passed since the start of this treatment; the left breast tumors continued to shrink, multiple brain metastases continued to remain stable, and left axillary lymph node involvement was not confirmed (Figure 2I-2L). Treatment comprising CDK4/6 inhibitors was continued.

## Discussion

A female patient with advanced breast cancer and brain metastases was treated with whole-brain radiotherapy and systemic chemotherapy, resulting in a reduced tumor volume. Additionally, CDK4/6 inhibitors and ET have maintained previous therapeutic effects, disease stability, and few side effects.

Metastases to the central nervous system are the most common neurological complications of systemic cancer [8], and breast cancer is the second leading cause of metastases in the central nervous system. Brain metastasis develops in 15% to 25 % of patients with breast cancer, including asymptomatic patients [1,9], during the clinical course and is associated with a poor prognosis.

In the human brain and spinal cord, the BBB is formed by endothelial cells that comprise the capillary walls and one of its important functions is the exclusion of macromolecules and water-soluble agents from entering the brain [10]. The BBB excludes most chemotherapeutic agents due to size restrictions and water solubility, including CDK4/6 inhibitors. However, in central nervous system metastases, the BBB is disrupted in various ways, and BBB permeability is unpredictable [4]. Although water-soluble agents do not penetrate the BBB, the combination of cisplatin and etoposide is effective for patients with brain metastases from breast cancer and lung cancer [11,12].

Approximately 70 % of patients with advanced breast cancer have HR+, HER2− disease, and ET is an effective and standard first-line therapy for metastatic disease [13]. Abemaciclib, which is a CDK 4/6 inhibitor, has demonstrated efficacy as a monotherapy and in combination with ET for advanced breast cancer [7,14,15]. The MONARCH-3 study was a phase III trial of abemaciclib plus a nonsteroidal aromatase inhibitor (anastrozole or letrozole) for postmenopausal women with advanced HR+ HER2− breast cancer without prior systemic therapy that demonstrated a progression-free survival and overall survival benefit [7]. Disease maintenance with hormonal treatment after a chemotherapy-induced response is a common strategy used to delay relapse and reduce treatment side effects [16]. The AMICA (Atrial Fibrillation Management in Congestive Heart Failure With Ablation) study evaluated the addition of the CDK 4/6 inhibitors to maintenance ET after the first palliative chemotherapy and found that this strategy offered promising efficacy and safety [17].

Most phase III studies, including the MONARCH-3 trial, excluded patients with brain metastases and disease progression sites that were not reported by other studies. In a rat orthotopic U87MG intracranial glioblastoma xenograft preclinical model, abemaciclib crossed the BBB and increased survival [18], indicating that abemaciclib may have activity in brain metastases arising from HR+ HER2− advanced breast cancer. An open-label, nonrandomized, phase II study found an intracranial objective response rate (primary endpoint) of 5.2% (3 patients) and an intracranial clinical benefit rate of 24.1% (11 patients); however, the primary endpoint was not achieved [19]. The potential therapeutic and preventive effects are not yet fully understood, and only a few case reports have been published [20]. In our case, after the tumor volume was sufficiently reduced by whole-brain irradiation and chemotherapy, the use of CDK4/6 inhibitors and ET as maintenance therapy resulted in long-term control of not only the primary lesion but also multiple brain metastases. However, this is a case report and treatment response may be influenced by multiple factors. While HR positivity is a potential predictor, the involvement of other mechanisms, including the abscopal effect, cannot be ruled out. This is the first case report of a patient who experienced long-term disease control and the prevention of new lesions with CDK4/6 inhibitor and ET.

## Conclusions

CDK4/6 inhibitors and ET resulted in beneficial long-term effects for a patient with breast cancer brain metastases. Breast cancer brain metastases are associated with a very poor prognosis, and systemic therapies are lacking. Novel treatment agents for breast cancer are expected to also have therapeutic effects on brain metastases. Yet treatment strategies specifically for breast cancer brain metastases are also necessary. However, more cases must be accumulated before these strategies can be developed.
